# Studies of a co-chaperone of the androgen receptor, FKBP52, as candidate for hypospadias

**DOI:** 10.1186/1477-7827-5-8

**Published:** 2007-03-07

**Authors:** Ana Beleza-Meireles, Michela Barbaro, Anna Wedell, Virpi Töhönen, Agneta Nordenskjöld

**Affiliations:** 1 Department of Molecular Medicine and Surgery, Karolinska Institutet, Stockholm, Sweden; 2 Department of Women and Child Health, Astrid Lindgren Children Hospital, Karolinska University Hospital, Stockholm, Sweden

## Abstract

**Background:**

Hypospadias is a common inborn error of the male urethral development, for which the aetiology is still elusive. Polymorphic variants in genes involved in the masculinisation of male genitalia, such as the androgen receptor, have been associated with some cases of hypospadias. Co-regulators of the androgen receptor start being acknowledged as possible candidates for hormone-resistance instances, which could account for hypospadias. One such molecule, the protein FKBP52, coded by the *FKBP4 *gene, has an important physiological role in up-regulating androgen receptor activity, an essential step in the development of the male external genitalia. The presence of hypospadias in mice lacking fkbp52 encouraged us to study the sequence and the expression of *FKBP4 *in boys with isolated hypospadias.

**Patients and methods:**

The expression of FKBP52 in the genital skin of boys with hypospadias and in healthy controls was tested by immunohistochemistry. Mutation screening in the *FKBF4 *gene was performed in ninety-one boys with non syndromic hypospadias. Additionally, two polymorphisms were typed in a larger cohort.

**Results:**

Immunohistochemistry shows epithelial expression of FKBP52 in the epidermis of the penile skin. No apparent difference in the FKBP52 expression was detected in healthy controls, mild or severe hypospadias patients. No sequence variants in the *FKBP4 *gene have implicated in hypospadias in our study.

**Conclusion:**

FKBP52 is likely to play a role in growth and development of the male genitalia, since it is expressed in the genital skin of prepubertal boys; however alterations in the sequence and in the expression of the *FKBP4 *gene are not a common cause of non-syndromic hypospadias.

## Background

Hypospadias is a common inborn error of the male genital development, consisting of a midline fusion defect of the male ventral urethra [1]. The urethral opening is ectopically located on the ventrum of the penis; and may be as proximal as the scrotum or perineum. This disorder occurs in approximately one out of every 300 male live births worldwide [2]; in Sweden, the incidence is 1.14 boys per 300 male live births according to the annual Swedish Malformation Registry. Despite being so common, its etiology is still largely unknown.

Male sexual differentiation is a process that depends on androgen action via the androgen receptor (AR). Androgens have a direct role in the fusion of the urethral folds [3,4]. Variants in the *AR *gene, such as CAG and GGN repeat polymorphisms, and in the 5-α reductase 2 gene (*SRD5A2*), which converts testosterone (T) to the more potent dihydrotestosterone (DHT) have been associated with hypospadias [5,6]. Androgen receptor defects have been shown to result in varying degrees of impaired masculinisation in XY individuals [7,8]; however this is thought to be infrequent in hypospadias [9,10]. Other factors may be more commonly implicated in its complex aetiology [11], such as environmental endocrine disrupters, or variants in other genes that are involved in the endocrine regulation of sexual differentiation.

AR, as a nuclear receptor, is subjected to a complex regulation by co-regulators and general transcription factors, which modulate androgen-targeted gene expression. In this context, hormone-resistance syndromes have already been attributed to disorders of co-regulatory proteins [12]. In 1999 New *et al *described two sisters with multiple partial hormone resistance, in which a co-activator defect has been proposed as the most likely underlying mechanism [13]. Likewise, Adachi *et al *in 2000 reported a patient presenting an androgen insensitivity phenotype, with normal *AR *gene; but lacking a protein that interacts with the AF-1 region of this receptor [14]. Furthermore, it has been suggested that dysfunction of one member of the large family of nuclear receptor co-regulators may produce mild hormone resistance syndromes, since compensatory mechanisms might be activated, a scenario compatible with isolated hypospadias [12]. The phenotype of the mice lacking fkbp52 (52KO), the FKBP52 orthologue, supports this concept.

FKBP52 is a tetratricopeptide repeat protein found in steroid receptor complexes, which directly control the transcriptional activity of such receptors [15-17]. To better assess the physiological importance of FKBP52, 52KO mice were generated. Surprisingly, 52KO males developed penile hypospadias with 100% penetrance [18,19]. Dysgenesis of anterior prostate and seminal vesicle, infertility and unilateral undescended testis was found in some mice. No abnormalities in testicular histology were observed in 52KO males. Gross defects in other organs and systems were ruled out. No alterations related to other steroid receptors were identified. The authors concluded that the phenotype of male 52KO mice is due to loss of Fkbp52-enhanced AR function [18,19], affecting its association with Hsp90, an important step in establishing and maintaining hormone binding ability [12-15].

Based on the 52KO mouse phenotype, mutations in *FKBP4 *may cause hypospadias in humans. In this report we examined the sequence and the expression of the *FKBP4 *gene in the skin of boys with different severities of hypospadias, and controls.

## Patients and methods

### 1. DNA analysis

#### Patients

Ninety-one boys with non-syndromic hypospadias, recruited through medical records in Sweden, were randomly selected. Of those, 64 were of Swedish origin; 21 of Middle-Eastern origin and 6 were from other nationalities. Patients with different degrees of severities of hypospadias were included: 30 cases had mild, 34 had moderate and 21 severe hypospadias; for the remaining patients, the severity was not possible to determine. The screening included 59 sporadic and 32 familial cases.

SNPs typing was performed in additional 242 non syndromic hypospadias patients, from the Swedish malformation registry, and 380 voluntary controls, among the Karolinska Hospital blood donors.

#### PCR and Sequencing

Genomic DNA was extracted from blood using a standard phenol/chloroform protocol. Primers (Table 1) flanking the exon/intron junctions were designed by the *Primer 3 *program and used to amplify the ten exons of the *FKBP4 *gene. PCR reactions were performed with DyNAzyme™ EXT DNA Polymerase (Finnzyme, Espoo, Finland) following manufacturer protocols (Table 2). After ExoSap-IT enzyme (USB Europe GmbH, Staufen, Germany) treatment the PCR fragments were sequenced on both direction using BigDye^® ^Terminator v3.1 kit (Applied Biosystems Warrington, United Kingdom) and analyzed in ABI Prism 3730 Sequencer. Sequence analysis was preformed with the program SeqScape v2.5 (Applied Biossystems).

**Table 1:** Primers used for PCR and sequencing.

	**Forward**	**Reverse**
**Exon1:**	GCAGAGGTGCTCAAGCCTC	CCTCGGTGCCTTAAACGAC
**Exon2:**	TCCCTTTATGTTCCCTCTGG	AACCATTCCTCCCTGAGCTT
**Exon3**:	TTCAGGAGCACTGTTTGAGC	GTCTCCAAGAAGCAGGAAGG
**Exon4:**	CTCTCGGATGAGAAAGATTGTG	GAGAACGGAAGTGTCTTGCC
**Exon5:**	GCTGATGGCATCCTTCCTC	CAAACAGCTGGTTCTACAATTCA
**Exon6–7:**	AGGAAATGGACAGGAAGCCT	TCTCAGTCACCAAGGGAAGG
**Exon8:**	AACCTCTTGTGGCCATGTGT	CAGACATGCTGGCAGCTCA
**Exon9:**	GGGTACCTTTGGAACCCAGT	ACAAAGAGCCCACAGTAGCC
**Exon10:**	CACCAGGCTTGGCCTATACA	GCCACCATCCAACCAGATAG

**Table 2:** PCR conditions. PCR reactions were performed with DynaEXT with standard protocols; exon 6 and 7 were amplified as one fragment.

	**Exons 2, 3, 4, 5, 9, 10**	**Exon 1**	**Exons 6–7,8**
DNA	25 ng	25 ng	25 ng
10×Buffer w.MgCl^2^	2,5 μl	2,5 μl	2,5 μl
10 mM dNTPs	0,5 μl	1 μl	0,5 μl
10 μM forward primer	1 μl	1 μl	1 μl
10 μM reverse primer	1 μl	1 μl	1 μl
DyNAzyme™ EXT	0,5 U	1 U	0,75 U
DMSO	0	1 μl	0
H_2_O	To 25 μl	To 25 μl	To 25 μl

#### Genotyping

Two SNPs in the *FKBP4 *gene were selected from the public databases: 1) the SNP rs1062478 (His > Arg), the only non-synonymous polymorphism in the gene with known frequency; and 2) the intronic SNP rs3021522 (C > G), the only polymorphism that was found by sequencing among the initially 91 screened patients. Typing was performed using a 5'-nuclease allele discrimination TaqMan assay with standard protocols (Applied Biosystems). The patients group was further divided into two sub-groups: 1) patients with Swedish ancestry (188); and 2) patients with non-Swedish ancestry (145). The samples were analyzed on an ABI 7900HT. Post analysis was performed with SDS software (Applied Biossystems) and Statistica 7.0.

### 2. Immunohistochemestry

#### Antibodies

Rabbit antibody anti-FKBP52 was provided by David Smith lab [18]. The secondary anti-rabbit antibody was obtained commercially (Santa Cruz and Vector).

#### Tissue preparation

Skin samples obtained during surgery from several hypospadias patients of different ages and severities were analysed for FKBP52 expression. Several age and ethnically matched healthy individuals were included as non-hypospadic controls, treated for other conditions. As positive control for the FKBP52 antibody we used human prostate [18]. Prostate tissue was obtained from a patient surgically treated for benign prostatic hyperplasia. The tissues were fixed for 5 to 7 hours in 4% formalin, washed four times in PBS four times and kept in 70% EtOH until paraffin embedding.

#### Immunohistochemistry

After dewaxing and rehydration, the paraffin sections were treated for antigen retrieval by heating at 98°C in 0.1 M Tris pH 9.0 for 20 minutes, after which the slides were allowed to cool to room temperature. Endogenous peroxidase activity was blocked by treating sections with 1 M H_2_O_2 _for 15 minutes in dark. To block nonspecific antibody binding, sections were preincubated in 10 mg/ml BSA (Sigma) containing 10% goat nonimmune serum (Vector) for 40 min. Affinity-purified polyclonal anti-FKBP52 was applied to the sections at a 1:100 dilution in 10 mg/ml BSA and allowed to incubate at 4°C overnight in a humid chamber. Control sections were incubated without primary antibody. The sections were incubated with the biotinylated secondary goat anti rabbit antibody (Vector) diluted in buffer containing 1% BSA and 10% goat serum followed by enzyme conjugate application (ABC, Vector) and chromogen development (AEC, Vector). All sections were counterstained with hematoxylin (Merck) for 15 sec before mounting in Kaisers glycerine gelatine (Merck). Images of immunostained tissued were captured using a Zeiss microscope. Background controls were performed similarly using only the secondary antibody.

In order to overcome the difficulties in quantifying immunohistochemestry, very standardized procedures were used to process the sections and the staining.

## Results

### Sequence analysis

Sequencing of the 10 exons and intronic/exonic borders, performed bi-directionally, did not reveal any coding sequence variant in the initially screened ninety-one patients. The intronic sequence variation rs3021522 (915C > G), in intron 6, was found in heterozygous form in five boys with hypospadias (2 Swedish and 3 Middle Easters). Analysis of this SNP and of an additional SNP rs1062478, His > Arg, in exon 4, was performed and analysed in 333 (including the initial 91) non-syndromic hypospadias patients and 380 controls. Genotyping yielded a 95% success rate. Allele and genotype frequencies are presented in table 3; totals represent only successful and unambiguous genotyping results. Differences in allele and genotype frequencies between patients and controls are not significant using Chi square and Fisher Exact tests, both when analysing all the patients and when only individuals with Swedish ancestry are included.

**Table 3:** SNP analysis: SNP typing performed in non-syndromic hypospadias patients and in a control population. The differences between cases and controls are not significant (p > 0,05). The frequencies on the whole group of patients does not differ from the frequencies on the Swedish sub-group (p > 0,05).

	**rs1062478 His > Arg**				**rs3021522 C > G**			
	
	**PATIENTS**		**CONTROLS**		**PATIENTS**		**CONTROLS**	
**GENOTYPES**	**g/g**	0	**g/g**	1	**g/g**	0	**g/g**	0
	**g/a**	1	**g/a**	0	**c/g**	16	**c/g**	24
	**a/a**	327	**a/a**	378	**c/c**	317	**c/c**	351

	**TOTAL**	328	**TOTAL**	379	**TOTAL**	333	**TOTAL**	375

	**g **(All patients)	1 (0.15%)	**g**	2 (0.26%)	**g **(All patients)	16 (2%)	**g**	24 (3%)
**ALLELES**	**g **(Swedish only)	1 (0.27%)			**g **(Swedish only)	8 (2,2%)		
	**a **(All patients)	655	**a**	756	**c **(All patients)	650	**c**	726
	**a **(Swedish only)	367			**c **(Swedish only)	366		

	**TOTAL **(Swedish only)	656 (368)	**TOTAL**	758	**TOTAL **(Swedish only)	666 (374)	**TOTAL**	750

### Immunohistochemistry

#### FKBP52 expression in human tissues

**Figure 1:**
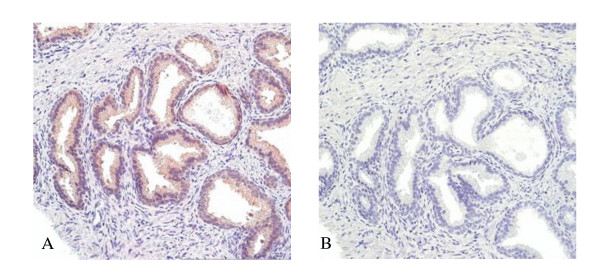
**FKBP52 expression in human prostate**. Prostate tissue was obtained from adult male surgically treated for benign prostatic hyperplasia. The tissue was fixed, paraffin embedded and sectioned before staining. Sections were immunostained with antibody specific for FKBP52 (A) and a consecutive section was immunostained with only secondary antibody for background detection (B). The strongest staining for FKBP52 is in the cytoplasm of ductal epithelial cells. (Sections photographed at 20× magnification).

To relate the mouse model more closely with potential human requirements for FKBP52, we looked at FKBP52 expression in human penile skin from hypospadias patients. Similarly to the mice, FKBP52 shows expression in luminal epithelial cells of human prostate (Fig. 1).

**Figure 2:**
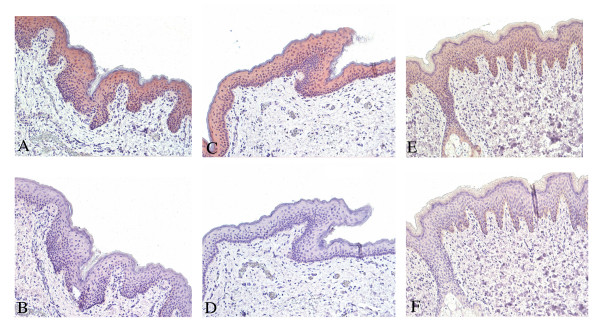
**FKBP52 expression in human pre-pubertal genital skin**. Foreskin samples from hypospadia patients and control individuals were obtained from surgery. The tissues were fixed, paraffinembedded and sectioned before staining protocols. The upper panel shows FKBP52 staining in mild hypospadia patient (A), severe hypospadia patient (C) and healthy control (E). The background controls for these samples using only the secondary antibody are seen in (B, D and F). The FKBP52 expression is predominantly cytoplasmic, localized in the epidermal region of the foreskin. Similar pattern and intensity are observed in healthy individuals and in hypospadia patients. (Sections photographed at 20× magnification).

FKBP52 immuno-staining is observed in the epidermis of prepubertal male genital foreskin, containing various cell types such as keratinocytes and melanocytes. Some staining is also seen in cells in the dermis, mostly consisting of fibroblasts and smaller blood vessels (Fig. 2). Though very similar expression pattern in various hypospadias sections, unspecific labeling from the primary polyclonal antibody cannot be absolutely ruled out. Similar to the prostate where FKBP52 and AR are co-expressed in luminal epithelial cells, AR is also observed in the epidermal region of the foreskin, mostly localized to nuclei whereas FKBP52 staining is predominantly cytoplasmic (not shown). We could not observe any apparent differences in the level of FKBP52 staining between mild or severe hypospadias patients. Besides, the healthy control individuals show similar pattern and intensity of FKBP52 expression in the epidermal and dermal regions.

## Discussion

Hypospadias is a common birth defect of the male genitalia for which the causes are still elusive. Currently, hypospadias is repaired surgically, constituting one of the most common surgeries performed on neonates. Although surgery may remain the therapy of choice, a better knowledge of the hormonal and molecular mechanisms of genito-urinary development may be the basis for preventive strategies reducing the incidence of this common malformation, and even therapeutic approaches.

Androgens have a clear role in the development of the male reproductive tract, acting via androgen receptor (AR). The complexity of the nuclear receptor regulation, including specific and unspecific co-regulators, opens a highly unexplored area of research in hypospadias; and has become an obvious target for genetic studies in individuals presenting signs of undervirilisation [12-14]. Such could be the case of FKBP52.

**Figure 3:**
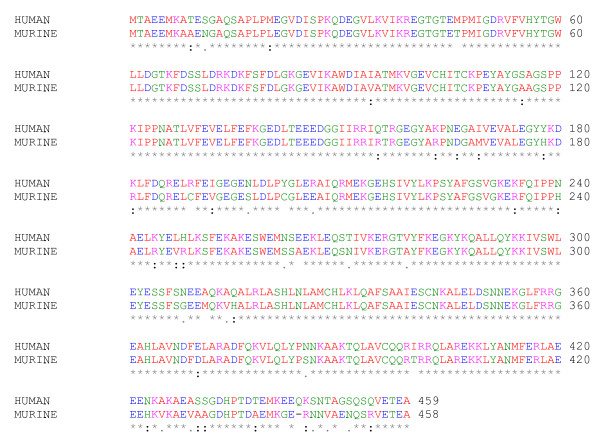
**Homology between the FKBP52 protein in humans (ref|NP_002005.1|) and the fkbp52 in mice (ref|NP_034349.1|)**. Alignments were performed with ClustalW revealing 89% homology between the two sequences.

Our results evidences that FKBP52, a co-regulator of AR, which plays a critical role in murine male external genital development, is expressed in genital skin of prepubertal boys. However, no obvious difference in the FKBP52 expression was apparent between hypospadias patients and healthy controls. Furthermore, the sequence analysis of the *FKBP4 *gene did not reveal any coding sequence variant. Despite the high homology (89%) between human and mice FKBP52 proteins, with conserved functional domains (Fig. 3), our results suggest that alterations on the human *FKBP4 *coding sequence and expression are not a common cause for non-syndromic hypospadias. These results may indicate that FKBP52 is less important for full virilisation of the male external genitalia in humans than in mice.

The amplification of the androgen activity is required for proper male external genital development in both human and mice. However differences in the regulation of the androgen pathway in the two species have already been described. It has been observed that the disruption of the 5-α reductase 2 gene (*Srd5a*) in mice does not induce any abnormal reproductive phenotype, while in humans, the presence of less active gene variants in its orthologue, *SRD5A2*, has been associated to hypospadias [9], infertility [20] and to various degrees of androgen insensitivity [21]. It is plausible that the androgen action is increased in men mainly by the conversion of testosterone to DHT by SRD5A2, while the mice external genital development may be more dependent on the combination of AR co-regulators such as FKBP52.

Indeed, the fine regulation of gene expression and hormonal activity are less well conserved between species then hormones and hormonal receptors [22]. Another possibility is that mutations in *FKBP4 *in humans result in other undermasculinisation phenotypes, which have not been the target of our study.

## Conclusion

The present report indicates that alterations in the sequence and in the expression of the *FKBP4 *gene are not a common cause of non-syndromic hypospadias. Furthermore it alerts to the importance of performing extrapolation from an animal model to humans with caution, despite the undeniable usefulness of model organisms, especially when it concerns the fine regulation of hormonal signalling, which may be species specific.
